# Factors affecting the understanding and retention of the informed consent among participants at an antiretroviral clinical trial in a resource limited setting

**DOI:** 10.1186/1745-6215-14-S1-P89

**Published:** 2013-11-29

**Authors:** Donald Salami

**Affiliations:** 1 Maryland Global Initiatives Corporation, Jos Plateau State, Nigeria; 2 University Of Liverpool(Laureate Online Education), Liverpool, UK

## 

The misunderstanding of the informed consent has become a major area of concern particularly for participants of clinical trials in the developing regions/countries. This study assessed the understanding and retention of the informed consent among participants in an anti-retroviral clinical trial been conducted in Nigeria; and also the myriad of social, cultural & economical factors affecting their understanding.

A structured questionnaire (QuIC) was administered to participants who signed consent to the lopinavir/ritonavir mono-therapy study, within the previous 12 months. The questionnaire assesses re-call of the basic elements of informed consent specified in federal regulations.

A total of 55 respondents completed the QuIC questionnaire. Majority age distribution was in the 30-49 age group (69.1%), 10.9% had no education, 32.7% were not employed, 69.1% were females. 74.5% didn’t know that the treatments and procedures in the clinical trial are not standard for their HIV management; 18.2% of respondents didn’t know that the trial carried additional risks and discomfort in comparison to standard treatments; 38.2% were unsure if they were offered alternatives to participation; 32.7% were unsure as to who will pay for treatment if they were injured or become ill as a result of participation in this clinical trial. None of the demographics were significantly associated with knowledge scores. In the uni-variate model, none of the socio-economic factors were independently associated with improved knowledge score.

Results suggest misunderstanding/deficiencies in certain important domain areas. This study reiterates the need for intensive studies in the area of therapeutic misconception as it has to do with HIV/AIDS preventive trials in developing countries.

